# Author’s Response to Peer Reviews of “Machine Learning–Based Prediction of COVID-19 Mortality With Limited Attributes to Expedite Patient Prognosis and Triage: Retrospective Observational Study”

**DOI:** 10.2196/34081

**Published:** 2021-10-15

**Authors:** Riccardo Doyle

**Affiliations:** 1 Stuart Ltd London United Kingdom

**Keywords:** COVID-19, coronavirus, medical informatics, machine learning, artificial intelligence, dimensionality reduction, automation, model development, prediction, hospital, resource management, mortality, prognosis, triage, comorbidities, public data, epidemiology, pre-existing conditions

*This is the author’s response to peer-review reports for “Machine Learning–Based Prediction of COVID-19 Mortality With Limited Attributes to Expedite Patient Prognosis and Triage: Retrospective Observational Study.”*

## Round 1 Review

### Reviewer DD

More detail is provided in the responses to individual comments [1], but for general context, to increase originality, the revised manuscript [2] now focuses more heavily on the impact of feature reduction on model performance rather than model performance as a standalone finding. The original reduction method, mutual information, is complemented by chi-square reduction, and comparisons between the impact of each were made, highlighting the need for different reduction methods to be tested as part of model tuning. Additional points were added to the *Discussion* stating that comparable models drawing from much richer feature sets performed comparably to our reduced ones and that large amounts of explanatory power can be captured by even a single variable, with the ultimate goal of reducing the number of variables, and consequently the tests and imaging, needed before models can be used in a hospital setting.

1. Mutual information was used due to the mixture of categorical and continuous variables, with a large presence of the former. A general equation for mutual information, which is the criterion used for feature selection, was provided. Variables were not binned but rather modelled through a k-nearest neighbors estimation approach; this was mentioned in the study, and the relevant source paper was cited for further detail. Software packages used (methods from Python’s sklearn library) were mentioned explicitly in the methodology.

2. Features are a subset/extraction of the original feature set, not a transformation/combination. A section was added to the *Results* section detailing the 7 most salient features selected via mutual information.

3. This would be a productive comparison; however, the reason it was not performed is due to data limitations. As outlined in the original paper, the 5121-patient data set has an extremely small proportion of patients affected by pre-existing conditions, meaning that keeping those features and training a 5121 patient model on age + comorbidities and comparing it to the full 212-patient data set would really simply be a comparison of the impact of age in the 5121-patient model against the full features in the 212-patient model, given that co-morbidity data is largely absent (and vastly underrepresented) in the 5121-patient data set. However, from other helpful revision comments, a feature importance table using mutual information was provided in the *Results* section; it shows that with the exception of fever, symptoms do not seem to play a high-importance role in prediction and do not feature in the top 7 explanatory variables.

Additionally, to further facilitate comparisons between data sets and feature reductions, only the 212-patient data set was retained in the study.

4. 95% confidence intervals were added to the result tables for all sensitivity, specificity, accuracy, and area under the curve (AUC) findings.

5. A paragraph has been added to the *Discussion* section briefly comparing the 7 features extracted in our study using mutual information to the most salient features from the proposed paper, finding substantial overlap, particularly with fever and pneumonia as high-value features.

6. The parameters used were reported in the *Results* section.

7. The date on which the data were accessed was added to the relevant data section in the methodology. Detailing the exact breakdown of samples in training and testing over multiple iterations of sample splitting and dimensionality reduction seems excessive, especially considering that the sample is small and retrievable and the methodology (3-fold cross-validation coupled with simple classifiers) is easily reproducible.

8. This was poor wording on the study’s end; it was intended to state that receiver operating characteristic (ROC) curves will be produced in order to obtain numerical AUC estimates, but the ROC plots were never meant to be graphically reported in the study. The original sentence was removed from the paper to avoid confusion.

### Reviewer EB

1. Noted; the suggestion [3] has been implemented. An in-depth review of existing equipment, public spending, and staff shortage limitations prior to the COVID-19 pandemic was provided with examples from around the world, with additional indicators of strain following the pandemic, as well as studies directly linking shortage of resources to worse patient outcomes, therefore justifying the need for better resource management. The primary management tool proposed in the study is the introduction of predictive modelling for better triage, providing potential benefits to “pre-allocation or local hospital transfer of life saving equipment, quantifying the need for further diagnostics or early treatment and directing limited staff attention and resources toward highest risk patients.” All condensed points in this response can be found in expanded form in the introduction of the study.

2. In the new *Discussion* section, a paragraph has been added regarding the real-world use cases of the models explored in the study.

3. Noted; a more streamlined and direct objective has been included in the new Abstract.

4. Noted; this has now been rectified.

5. Noted; this has now been rectified.

6. This refers to the Area Under the Curve, and a footnote has now been added.

## Abbreviations

AUC: area under the curve
ROC: receiver operating characteristic
